# Effects of different anti-caries agents on microhardness and superficial microstructure of irradiated permanent dentin: an in vitro study

**DOI:** 10.1186/s12903-019-0815-4

**Published:** 2019-06-14

**Authors:** LiLing Wu, Kun Geng, QingPing Gao

**Affiliations:** 1 Center of Stomatology, Xiangya Hospital Central South University, Changsha, 410008 Hunan Province China; 2 Department of Stomatology, ZhongShan Hospital Xiamen University, Xiamen, 361004 China

**Keywords:** Radiotherapy, Permanent dentin, Infiltration resin, Casein phosphate polypeptide-amorphous calcium phosphate (CPP-ACP)

## Abstract

**Background:**

To compare different anti-caries agents on microhardness and micromorphology of irradiated permanent dentin in vitro, and try to find the most effective agent to prevent radiation-dentin-destruction.

**Methods:**

A total of 120 dentin samples were prepared from 60 human teeth and randomly divided into 8 groups (*n* = 15), [ (1)] blank control [2]; irradiation control [3]; irradiation+ fluoride [4]; irradiation+ casein phosphate polypeptide-amorphous calcium phosphate (CPP-ACP) [5]; irradiation+ CPP-ACP+ fluoride [6]; irradiation+ infiltration resin [7]; irradiation+ infiltration resin+ fluoride [8]; irradiation+ infiltration resin+ CPP-ACP. Seven samples of each groups were chosen randomly for microhardness test and eight for scanning electron microscope observation. Results: A decrease of microhardness (*P* < 0.05) and an obvious morphological change were presented on dentin surface after radiotherapy. After applications of anti-caries agents, the morphological destructions were effectively restored. The infiltration resin plus fluoride group (56.00 ± 4.02 Kg/mm^2^), infiltration resin plus CPP-ACP group (56.05 ± 3.69 Kg/mm^2^), infiltration resin group (54.70 ± 4.42Kg/mm^2^) and CPP-ACP plus fluoride group (53.84 ± 6.23Kg/mm^2^) had the highest dentin microhardness value after radiotherapy, and no statistically significant difference were found between them.

**Conclusions:**

Infiltration resin, CPP-ACP, fluoride and their pairwise combination can effectively prevent radiation-dentin-destruction. Among them, infiltration resin with CPP-ACP, infiltration resin with fluoride, CPP-ACP with fluoride, and infiltration resin have the most protective effects on irradiation-dentin-destructions.

## Background

Radiation related-caries (RRC) is one of the most common oral complications with the incidence of 90% in head and neck cancer (HNC) patients after radiotherapy [1]. There is evidence that the co-morbidity of radiotherapy on teeth is one of the important pathogenic factors for RRC, including changes in microstructure and mechanical properties of dental hard tissues [2]. Because of the rapid progression of RRC and the difficulty of controlling, RRC is also known as ‘rampant caries’, whose treatment is mainly focus on prevention in clinical.

After half a century of researches, the benefits of fluorides have been firmly established [3]. Fluorides, which are currently thought to be an effective anti-caries agent used by form of mouthwashes, dentifrices, gels, varnishes, prevent caries by inhibiting demineralization and promoting remineralization [4]. Fluorides are always used as positive control materials whenever new remineralization agents need to be tested [4]. For the last decade, a novel product casein phosphopeptide-amorphous calcium phosphate (CPP-ACP), which is a nanocomplex derived from milk protein and usually used for the management of incipient caries, has become commercially available [5]. Casein phosphopeptide (CPP), a non-cytotoxic bioactive peptide, combines with amorphous calcium phosphate (ACP) to form a CPP-ACP soluble complex, which is capable of affecting the demineralization-remineralization processes [6]. The anti-cariogenic properties and remineralizing effects of CPP-ACP have been demonstrated in animal laboratory [7], in vitro studies [8] and human in situ experiments [9]. However, the reported anti-caries efficacy of CPP-ACP in comparison with traditional fluorides was controversial [10]. Somasundaram [11] et al. evaluated the protective effects of CPP-ACP paste and fluoride toothpaste on demineralized caries lesions and the results showed that CPP-ACP significantly reduced the depth of lesions with 20.22% more than that of fluoride toothpaste. Though Lata [12] and Oliveira [13] et al. found that the effects of CPP-ACP on the remineralization of caries lesions were weaker than that of fluorides. Besides, it is arguable whether fluorides and CPP-ACP have combined effects on irradiation-caries [14, 15].

Resin infiltration technique, a promising micro-invasive approach to prevent demineralization, may mechanically stabilize the demineralized lesions and was considered as an alternative treatment for fluorides and CPP-ACP [16]. In addition, infiltration resin can significantly improve the microhardness of demineralized dental hard tissues and reduce the loss of minerals [17]. It was reported that infiltration resin had combined effects with CPP-ACP and fluoride in preventing early caries [18, 19]. To the best of our knowledge, whether this anti-cariogenic effect will be the same when infiltration resin applied alone or in combination with CPP-ACP and fluoride to irradiated dentin is still unknown.

Therefore, the purpose of this study was to compare, by micro indentation hardness test and scanning electron microscopy (SEM), the in vitro ability of different anti-caries agents to prevent damages of dentin caused by radiotherapy. The null hypothesis tested was that different anti-cariogenic methods would not influence the micromorphology and microhardness of irradiated permanent dentin.

## Methods

### Sample preparation

Sixty healthy human permanent posterior teeth were collected from Center of Stomatology of Xiangya Hospital of Central South University after suitable approval from the Human Research Ethics Committee of the same institution (Number: 2017121128). The teeth were extracted for orthodontic reasons and none of them had any caries, restorations, defects or enamel hypoplastic. The teeth were thoroughly washed and ultrasonically scaled to remove plaque and calculus, and polished with rubber prophylaxis cup at a low-speed handpiece with a mixture of non-fluoridated oil-free pumice and water, then stored in physiological saline solution at a temperature of 4 °C (DW-25 W322, China) and used not more than 3 months post-extraction [20].

The teeth were cleaned and had their roots and removed at the cementum-enamel junction (CEJ) using a high-speed diamond disc (MANI, Japan) under cooling-water. The pulp tissue was then removed. Crowns were sectioned longitudinally into two halves in the mesiodistal direction, yielding 120 specimens. Occlusal enamel sections of all specimens were removed from crowns using a high-speed diamond disc to expose the dentin surface, which was considered as tested surface and ground with an increasing sequence of silicon carbide (SiC) paper (1500, 2000, 2500, 3000, 5000 and 7000 grit, STARCKE, Germany) for 60s each under running water to produce flat dentin surface [21]. The exposed dentin surface was the surface dentin layer about 1~2 mm below enamel. Between each polishing stage, samples were cleaned using physiological saline solution in an ultrasonic cleaner (JP-010 T, China) for 8 min. These 120 samples were divided into eight groups by a random number table until the groups reached a total of 15 dentin samples each. All the dentin samples were embedded with medical red wax and exposed the tested surface, which was covered with two layers of acid-resistant nail polish, leaving a 3 mm × 3 mm window exposed for microhardness and SEM test.

### Irradiation procedure

The radiation dose was calculated based on a mean that were applied on 166 HNC patients collected from Oncology Department of Xiangya Hospital [22]. The radiotherapy protocols consisted of a total of 35 fractions equal to 68.25 Gy, with 1.95Gy exposure per day applied 5 days per week, during 7 weeks with X-rays from a linear accelerator (Varian, Clinac 23EX, America) in the Department of Radiotherapy at Xiangya Hospital.

### Tested groups and interventions

For each group, the open window area was treated with different protocols. All dentins were submerged in physiological saline solution and the normal saline was changed every day. In between the radiation cycles, the dentins were stored in physiological saline solution at a temperature of 37 °C (DW-25 W322, China). The researcher who conducted the interventions of 8 experimental groups was not participated in the statistical analysis.

Group1 (Control group): The dentins were not treated with irradiation or any anti-caries agents.

Group2 (Irradiation): The dentins were irradiated but not treated with any anti-caries agents.

Group3 (Irradiation+ Fluoride): Every-time before irradiation, fluoride varnish (3 M Clinpro™ White Varnish) was applied on dentins and left for 3 min then cleaned with normal saline. When the maximum cumulative radiation dose of 68.25Gy was delivered, fluoride varnish was continued applied 5 days per week for 4 weeks.

Group4 (Irradiation+ CPP-ACP): Every-time before irradiation, CPP-ACP (GC Tooth Mousse™, Tokyo, Japan) was applied on dentins and left for 3 min then cleaned with normal saline. When the maximum cumulative radiation dose of 68.25Gy was delivered, fluoride varnish was continued applied 5 days per week for 4 weeks.

Group5 (Irradiation+ Fluoride+ CPP-ACP): Every-time before irradiation, fluoride varnish was applied on dentins and 5 min later CPP-ACP was used. When the maximum cumulative radiation dose of 68.25Gy was delivered, fluoride varnish and CPP-ACP was continued applied 5 days per week for 4 weeks.

Group6 (Irradiation+ Infiltration resin): When the maximum cumulative radiation dose of 68.25Gy is reached, in accordance with the manufacturer’s instructions [18], the dentins were etched with 15% HCL gel for 120 s, rinsed for 30 s, and dehydrated with 100% ethanol for 30 s. Infiltration resin (Icon™, DMG, Hamburg, Germany) was applied with a micro brush for 3 min, excess material was gently removed by air blowing and flossing, then the resin was light-cured for 40 s. Infiltration resin was applied a second time for an additional 1 min, light curing for 40s.

Group7 (Irradiation+ Infiltration resin+ Fluoride): When the maximum cumulative radiation dose of 68.25Gy was delivered, infiltration resin was applied accordance to the manufacturer’s instructions then fluoride varnish was used 5 days per week for 4 weeks.

Group8 (Irradiation+ Infiltration resin+ CPP-ACP): When the maximum cumulative radiation dose of 68.25Gy was delivered, infiltration resin was applied accordance to the manufacturer’s instructions then CPP-ACP was used 5 days per week for 4 weeks.

At the end of all interventions, five dentins of each groups were chosen for scanning electron microscope observation. Before the first-time radiation and after all interventions, ten dentins were used for microhardness test.

### SEM analysis [23]

After the cumulative dose of radiotherapy and another 4 weeks of remineralization, five dentins of each groups were chosen for scanning electron microscope observation. Observation analyses with SEM were performed by a senior researcher who was experienced, calibrated and blinded regarding the experimental groups. Eight dentins from each group were fixed in 2% glutaraldehyde in cacodylate buffer for 2 h and were dehydrated in a series of increasing ethanol concentrations (30, 50, 75, 95, and 100%) for 10 min. Subsequently, specimens were sputter-coated with gold in a vacuum metallizing machine (SCD500, BAL-TEC, Switzerland) and examined with a scanning electron microscope (JSM-6490LV, Neptune Tex’s HP, Japan). Two images were obtained of each dentin samples at two magnifications (*2000 and *5000).

### Microhardness testing [24]

Microhardness testing was performed by a senior researcher who was blinded to the sample groupings. Surface hardness of 7 dentins from each group was determined using a microhardness tester with a Vickers diamond indenter (HMV-2 T Microhardness Tester, Shimadzu, Kyoto, Japan). The baseline surface microhardness (SMH_0_) and microhardness post-interventions (SMH_1_) of all dentins were measured.

### Statistical analysis

The researcher who conducted the statistical analysis was masked to the experimental groups. All tests employed *α* = 0.05 as level of significance and statistical analysis was performed with IBM SPSS Statistics Version 22 for Windows. Student’s *t* test was used to compare the microhardness pre-and post-irradiation within groups. One-way analysis of variance (ANOVA) was used for comparison between groups. LSD-*t* test was used for pairwise comparison between groups when ANOVA test was significant.

## Results

### SEM analysis

**Fig. 1:**
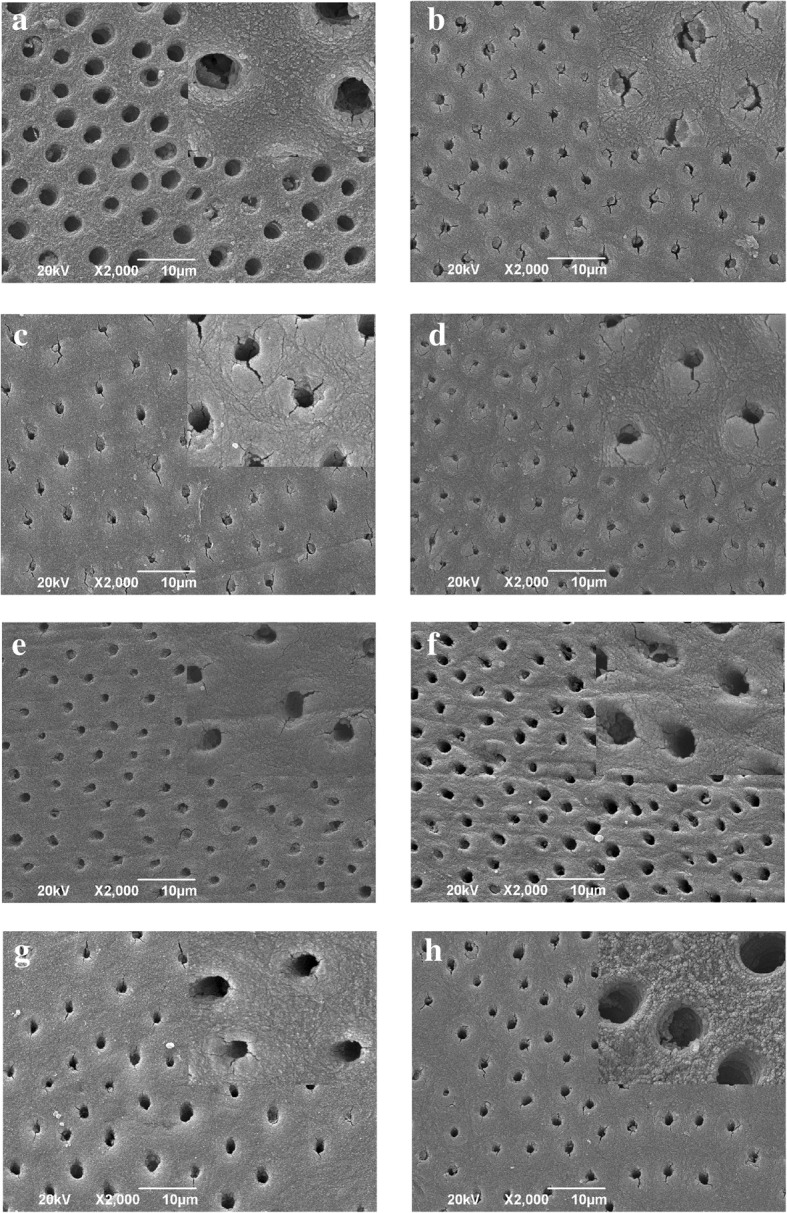
Representative SEM photographs of dentin surface from different groups (*2000, insets *5000): **a**. Control group; **b**. Irradiation group; **c**. Irradiation + Fluoride; **d**. Irradiation + CPP-ACP; **e**. Irradiation + Fluoride + CPP-ACP; **f**. Irradiation + Infiltration resin; **g**. Irradiation + Infiltration resin + Fluoride; **h**. Irradiation + Infiltration resin + CPP-ACP

From the initial 120 dentin samples used in this study, none of them was rejected or lost during and after processing. Figure 1 showed the representative SEM photographs (*2000, insets *5000) of dentin surface from different groups. SEM images showed that in sound dentin, it is possible to note normal organization of dentin, showing a uniform size and shape of dentin tubules, with homogeneous peritubular and intratubular dentin (Fig. 1a). The well-defined structure of dentin was destroyed after irradiation exposure with 68.25 Gy, showing dentin tubules shrunk, or even completely closed, and most of them blocked. The peritubular and intratubular dentin were irregular, accompanied by cracks and uneven surface (Fig. 1b). The moderate destructions of dentin micromorphology were found in fluoride group (Fig. 1c) and CPP-ACP group (Fig. 1d). SEM photographs showed that partial dentin tubules were blocked and the cracks in peritubular and intratubular dentin reduced. Following application of fluoride with CPP-ACP (Fig. 1e), infiltration resin (Fig. 1f), infiltration resin plus fluoride (Fig. 1g), and infiltration resin plus CPP-ACP (Fig. 1h), the micrographs of dentin were slightly changed. The surfaces were well-distributed and smooth and only a few shallow pits and cracks around tubules were observed. The peritubular and intratubular dentin were homogeneous.

### SMH analysis

The SMH values of dentin for all groups pre-and post-irradiation were presented in Table 1. The results of Student’s *t* test pre-and post-irradiation intra-groups showed that irradiation significantly reduced the SMH of dentin (*P*<0.05). One-way ANOVA analysis showed that no significant difference was found in dentin SMH between 8 groups pre-irradiation (Table 2, *P*>0.05). However, a statistically significant difference was found in SMH post-irradiation between the groups (Table 3, *P*<0.05). Six different anti-caries agents (three applications alone and three pairwise combined applications) significantly increased the SMH of irradiated dentin (*P*<0.05). However, they cannot restore the SMH of irradiated dentin to non-irradiation level. The order of dentin SMH after radiotherapy from the highest to the lowest was as follows: G1(control group) > G8(infiltration resin+ CPP-ACP) ~G7(infiltration resin+ fluoride)~G6(infiltration resin)~G5(fluoride+ CPP-ACP) > G4(CPP-ACP)~G3(fluoride) > G2(irradiation group). The SMH values of G5-G8 were the highest among 7 irradiated dentin group, and LSD-*t* test showed that no statistically significant difference was detected between them (Table 3; *P*>0.05). G3 and G4 groups were not statistically different as well (Table 3; *P*>0.05).

**Table 1:** Microhardness value of permanent dentin treated with different anti-caries agents, and results of Student’s *t* test pre-and post-irradiation intra-groups (^*−*^*χ* ± *S*, *n* = 7, MHV)

Groups	SMH_0_ (Kg/mm2)	SMH_1_ (Kg/mm2)	*t*	*P*
G1	65.36 ± 1.89	65.72 ± 2.40	−0.687	0.52
G2	65.42 ± 1.68	39.18 ± 6.35	9.662	0.00*
G3	65.36 ± 3.22	46.92 ± 4.88	7.122	0.00*
G4	64.90 ± 2.86	47.07 ± 6.39	5.765	0.00*
G5	65.25 ± 1.52	53.84 ± 6.23	5.830	0.00*
G6	65.13 ± 3.19	54.70 ± 4.42	4.772	0.00*
G7	65.27 ± 1.98	56.00 ± 4.02	7.299	0.00*
G8	65.88 ± 5.50	56.05 ± 3.69	8.159	0.00*

*means that there was significant difference in the dentin microhardness pre- and post-irradiation intra-group (*P*<0.05). *SMH* surface microhardness

**Table 2:** Microhardness value of permanent dentin treated with different anti-caries agents, and results of One-way ANOVA analysis pre-and post-irradiation between-groups (^−^*χ* ± *S*, n = 7, MHV)

Groups	SMH_0_ (Kg/mm^2^)	SMH_1_ (Kg/mm^2^)	*F*_1_	*P*_1_	*F*_2_	*P*_2_
G1	65.36 ± 1.89	65.72 ± 2.40	0.06	1.00	17.90	0.00*
G2	65.42 ± 1.68	39.18 ± 6.35				
G3	65.36 ± 3.22	46.92 ± 4.88				
G4	64.90 ± 2.86	47.07 ± 6.39				
G5	65.25 ± 1.52	53.84 ± 6.23				
G6	65.13 ± 3.19	54.70 ± 4.42				
G7	65.27 ± 1.98	56.00 ± 4.02				
G8	65.88 ± 5.50	56.05 ± 3.69				

*F*_1_, *P*_1_: One-way ANOVA analysis in dentin SMH between 8 groups before irradiation. *F*_2_, *P*_2_: One-way ANOVA analysis in dentin SMH between 8 groups after irradiation. *SMH* surface microhardness. *means that there was significant difference in dentin SMH post-irradiation between 8 groups (*P*<0.05)

**Table 3:** Microhardness value of permanent dentin treated with different anti-caries agents, and results of LSD-*t* pairwise comparison test post-irradiation between-groups (^−^*χ* ± *S*, n = 7, MHV)

Groups	SMH_1_ (Kg/mm^2^)	*P*_1_	*P*_2_	*P*_3_	*P*_4_	*P*_5_	*P*_6_	*P*_7_	*P*_8_
G1	65.72 ± 2.40	–	0.00	0.00	0.00	0.00	0.00	0.00	0.00
G2	39.18 ± 6.35	0.00	–	0.01	0.01	0.00	0.00	0.00	0.00
G3	46.92 ± 4.88	0.00	0.01	–	0.96	0.01	0.01	0.00	0.00
G4	47.07 ± 6.39	0.00	0.01	0.96	–	0.01	0.01	0.00	0.00
G5	53.84 ± 6.23	0.00	0.00	0.01	0.01	–	0.75	0.42	0.41
G6	54.70 ± 4.42	0.00	0.00	0.01	0.01	0.75	–	0.63	0.62
G7	56.00 ± 4.02	0.00	0.00	0.00	0.00	0.42	0.63	–	0.98
G8	56.05 ± 3.69	0.00	0.00	0.00	0.00	0.41	0.62	0.98	–

*P*_n_ means that results of LSD-*t* test between Gn and other 7 groups (n = 1~8). SMH: surface microhardness. *means that there was significant difference in dentin SMH post-irradiation between-groups (*P*<0.05)

## Discussion

The aim of this study was to compare the preventive effects of different anti-caries agents against the development of radiation-dentin-destructions by simulating the influences of irradiation on the micromorphology and the mechanical properties of dentin in vitro. It is indeed that the radiation affected the structural and mechanical properties of dentin. Infiltration resin, CPP-ACP and fluoride, whether used alone or in combination with each other, can effectively restore the destruction of dentin surface caused by irradiation and increase its surface microhardness. Among them infiltration resin and its combination with CPP-ACP and with fluoride, as well as the combination of CPP-ACP and fluoride had the most remarkable effects on the prevention of radiation-dentin-destructions. The null hypothesis was rejected.

In clinical, head and neck cancers (HNC) are commonly occurred in elderly population, whose dentins are always directly exposed to oral cavity due to severe masticatory attrition. In order to better simulate the tooth characteristics of irradiated HNC patients, occlusal enamel sections of samples were removed from crowns and the dentin surfaces were exposed to treatment. Mineral content varies at different levels of dentin. In the surface layer of dentin, mineral content was more than middle and inner layers of dentin as there are structural changes in all the levels of the dentin. In our experiment, we studied the surface layer of dentin. SEM is widely used to observe microstructural changes in the surface of dental hard tissues after being treated with different anti-caries agents [25]. Microhardness is one of the important physical properties of tooth surface, which reflects the ability to resist plastic deformation and persistent indentation. Featherstone [26] et al. found that a linear correlation was observed between microhardness and dental mineral content. Therefore, the determination of dentin surface microhardness and SEM has become important measures and indexes to estimate the demineralization and remineralization of dental hard tissues in the studies of caries researches.

In our study, only the microhardness values before and after the interventions were measured, which was carried out according to other studies. Studies from Barros et al. [27] and Reed et al. [28] have only measured the experimental parameters two times (before and after interventions treatment), and they did not make a continuous curve graph. In our study, a direct irradiation group was set up. By comparing the direct irradiation group with the different remineralization agents’ groups, the protective effect of the remineralization agents on the irradiated tooth could be found intuitively. The purpose of our study was not to observe the dose-response effect of remineralization agents and radiotherapy dose on dental hard tissue, but to observe whether remineralization agents had effects on irradiation-dentin-destructions. Therefore, our study only measured the microhardness twice before and after the experimental interventions.

Our results showed that radiation had a significant influence on the micromorphology of dentin, resulting in the appearance of cracks on the dentin surface and the occlusion of dentin tubules. The destruction of peritubular and intratubular dentin was also observed, which squeezes the dentin tubules to obstruct them [29]. These microstructure changes were important factors for radiation-caries in HNC patients [30]. The Vickers microhardness of dentin is generally in the range of 58.23~73.56Kg/mm^2^ in this study. After 68.25Gy dose of irradiation, decrease in microhardness values accompanied by superficial morphology alterations were found in dentin surface, which was consistent with previous studies [31, 32]. Fränzel [31] et al. was even found that the microhardness of dentin post-radiotherapy was 61% lower than that of pre-radiotherapy.

Velo [33] et al. believes that radiation exposure changes the composition and structure of human dentin and adversely affects its microhardness. On the one hand, radiation causes the oxidation of water molecules to produce hydrogen free radicals and highly reactive oxygen species, which results in the denaturation of organic components [31, 34]. On the other hand, the interaction of organic matrix and apatite crystal in dentin promotes the electrostatic binding, which is formed between carboxylate of collagen side chain and phosphate through calcium ion. Radiation induces decarboxylation of carboxylate on the side chain of collagen, and loss of acidic phosphates resulting in the formation of new calcium ion-carboxylic acid complex. In addition, the microcracks in hydroxyapatite were occurred because of the decrease of apatite crystals-organic matrix interaction and the increase of carbon dioxide production, resulting in rough dentin surface and decreased microhardness [35]. In addition, radiotherapy increases the sensitivity of dental hard tissues to demineralization after being irradiated [30]. Studies [31, 34] also declared that the deleterious damages of irradiated dental hard tissues were similar to the demineralization process of teeth. These structural defects will increase the dryness and fragility of dentin [32], weaken their mechanical resistance and decrease their microhardness after radiotherapy, which may be related to the rapid development of radiation-caries.

It is necessary to prevent early demineralized dentin and promote its remineralization in time, which is not only a therapeutic measure, but also a preventive measure for further subsurface demineralization. Therefore, early detection and remineralization of demineralized lesions through effective applications of anti-caries agents are important methods to prevent radiation-destructions in irradiated HNC patients. Different preparations in this study were applied on irradiated dentin and we have noted that each treatment was to some extent effective. In this study, infiltration resin was used for the first time in preventing radiation-dentin-destructions and achieved significantly curative effects. When applying infiltration resin to prevent the demineralization of dental hard tissue, it is often used after demineralization process [36]. After light curing, the resin-porous hydroxyapatite complex was formed, which occludes micro-porosities within the demineralization lesion, forming a diffusion barrier to prevent further demineralization. Hence, in this study, the application of infiltration resin was used after the cumulative radiation dose delivered.

When the irradiated dentin was treated with one of three anti-caries agents alone, the effect of infiltration resin on the micromorphology and microhardness was the strongest, which was consistent with previous studies reported that the effects of infiltration resin on the surface microhardness of artificial caries was better than that of fluoride [37] and CPP-ACP [38] due to the high permeability of infiltration resin. The remineralized layer formed by fluoride or CPP-ACP can seal the dentin surface with a very shallow permeation depth. However, infiltration resin can penetrate into the inner space of demineralized dentin. Its average penetration depth is 516.8 μm, and the deepest depth is 973 μm, forming a diffusion barrier inside dentin [36]. Therefore, the structure of deep demineralized dentin was significantly restored, and the mechanical properties of dental hard tissues were strengthened. Shaik [39] et al., who studied the remineralizing potential of CPP-ACP and resin-infiltration by the quantitative evaluation of mineral gain (Ca:P), found that CPP-ACP performed better than resin-infiltration in remineralizing the artificial carious lesions, which was not in agreement with our study. However, just mineral content ratio (Ca:P) of the specimens were recorded in Shaik’s study, surface microhardness and superficial morphology were not evaluated.

In this study, the protective effects on microhardness and micromorphology of irradiated dentin in infiltration resin group were as effective as the simultaneous use of infiltration resin plus CPP-ACP, infiltration resin plus fluoride and fluoride plus CPP-ACP. The effects of all these four groups were significantly greater than using fluoride or CPP-ACP monotherapy. There have been reports that inhibition of progression in incipient caries lesions was significantly effective after treatment of resin infiltration and fluoride varnish than that of fluoride monotherapy [40, 41]. Shi Chen [19] et al. found that the combined treatment of infiltration resin and CPP-ACP better improved the effects both in remineralization and inhibition of demineralization than CPP-ACP monotherapy. However, there was no significant difference between infiltrant and its combined effects with CPP-ACP or with fluoride in our study. This may due to the permanent occlusion of micropores and cavities by infiltration resin, which makes fluoride and CPP-ACP unable to penetrate into micropores. To some extent, the remineralized ions were prevented from entering into the demineralized lesions to form a hard hydroxyapatite crystal structure. Therefore, infiltration resin plays a leading role in the combined use with fluorides or CPP-ACP for promoting the remineralization of irradiated dentin.

In this study, fluoride and CPP-ACP can effectively promote dentin remineralization and have synergistic effects. Combined application of fluoride and CPP-ACP effectively reduces the progression of radiation-dentin-destructions than their monotherapy. Studies [14, 42] have demonstrated that simultaneous use of CPP-ACP with fluorides achieved significantly clinical advantages over CPP-ACP or fluorides in reducing the progression of radiation-caries in patients with nasopharyngeal carcinoma, which was in accordance with our study. In the presence of fluoride, CPP-ACP mixed with fluoride ions to form a new complex: casein phosphopeptide-amorphous calcium fluoride phosphate (CPP-ACFP), which produces more effectively remineralizating components than fluorides and CPP-ACP [43]. Hay [44] et al. compared effects of CPP-ACP and fluoride on preventing radiation-caries in HNC patients. It was showed that both of them could effectively prevent the formation of radiation-caries, but there was no significant difference between them. The simultaneous application of CPP-ACP with fluorides has synergistic effects in promoting remineralization of caries and inhibiting demineralization as well. Therefore, the current study argues that CPP-ACP could be used in clinical in collaboration with fluorides rather than applied as an alternative to fluorides.

The appearance of dental fluorosis and osteofluorosis in the process of application limited the clinical use of fluorides to some extent. Hence, it should be cautious to apply fluorides for treating caries lesions. Several studies have shown that no severe side effects have been found when evaluating the clinical safety of CPP-ACP [45]. During the two-year follow-up period, Morgan [45] et al. assessed the side effects between CPP-ACP group and placebo control group and no statistically significant differences were found in the incidence of diarrhoea, headache and nausea between groups. CPP-ACP were derived from milk protein casein, with the results that people with milk allergies are not allowed to use CPP-ACP. Similarly, no side effects associated with infiltration resin have been observed [46]. Infiltration resin and CPP-ACP have great clinical safety and are effective for remineralization of irradiated dentin, so both of them can be widely used in clinical as preventive agents for radiation-destructions.

This study was an in vitro experiment mainly based on permanent dentin, which cannot completely simulate the actual oral environment of irradiated HNC patients. As to the mechanical properties of dental hard tissues, Vickers microhardness was selected. However, other parameters such as fracture resistance, ultimate tensile strength and elastic modulus, needed to be further studied. In our experiments, no scoring system was used when recorded the structural changes. Since no uniform evaluation system of dental hard tissue demineralization was used for scanning electron microscopy (SEM) observation at present, and the microscopic changes of dentin surfaces could be observed directly from the naked eyes. The observation of SEM photographs in our experiment was based on the naked eyes. However, direct naked-eyes observation of SEM results may be prone to heterogeneity and poor reproducibility to a certain extent. In our experiments, in group 6, 7 and 8 the Infiltration resin was used. We chose to apply Infiltration resin after the cumulative dose of radiotherapy for the following reasons: Infiltration resin is a new agent applied for incipient caries and it was first-time applied on irradiated dental tissues. Infiltration resin is light-cured resin. At present, DMG company (Germany) recommends that the time point for infiltration-resin-application was after tooth demineralization. It is not used for prevention of non-demineralized teeth. However, it may change the standardization of the methodology.

Therefore, more in vitro, in vivo studies and clinical trials are needed to further demonstrate the remineralizing potential and long-term anti-caries efficacy of infiltration resin, CPP-ACP, fluoride and their pairwise combined application for the radiation-destructions.

## Conclusions

Based on the data from this in vitro study, we make the following conclusions [1]: The exposure of 68.25Gy irradiation affects micromorphology and microhardness of permanent dentin [2]. To some extent, infiltration resin, CPP-ACP, fluoride and their pairwise combined applications can effectively prevent radiation-dentin-destructions. Among them, infiltration resin with CPP-ACP, infiltration resin with fluoride, CPP-ACP with fluoride, and infiltration resin monotherapy have the most protective effects on irradiated-dentin-destructions.

## Data Availability

All the data generated or analysed during this study are included in this published article.

## Abbreviations

ANOVA: One-way analysis of variance
CEJ: cementum-enamel junction
CPP-ACFP: casein phosphopeptide-amorphous calcium fluoride phosphate
CPP-ACP: casein phosphate polypeptide-amorphous calcium phosphate
HNC: head and neck cancer
RRC: Radiation related-caries
SEM: scanning electron microscopy
SMH: surface microhardness
